# Implication of agricultural practices in the micro-geographic heterogeneity of malaria transmission in Bouna, Côte d’Ivoire

**DOI:** 10.1186/s12936-023-04748-3

**Published:** 2023-10-17

**Authors:** Obo Armel-Hermann Beke, Serge-Brice Assi, Akedjro Paul Harvey Kokrasset, Kacou Jean Denis Dibo, Méa Antoine Tanoh, Mathias Danho, Franck Remoué, Guibehi Benjamin Koudou, Anne Poinsignon

**Affiliations:** 1 National Malaria Control Programme, Abidjan, Côte d’Ivoire; 2 Swiss Center of Scientific Research, Abidjan, Côte d’Ivoire; 3 Institut National Polytechnique Houphouët Boigny, Yamoussoukro, Côte d’Ivoire; 4 Institut National de Santé Publique / Institut Pierre Richet, Bouaké, Côte d’Ivoire; 5 MIVEGEC, University of Montpellier, IRD, CNRS, Montpellier, France

**Keywords:** Malaria, Hotspot, Microgeographic heterogeneity, Agriculture practices, *Anopheles* exposure

## Abstract

**Background:**

Wetlands and irrigated agricultural crops create potential breeding sites for *Anopheles* mosquitoes, leading to a heterogeneity in malaria transmission. In agricultural areas, heterogeneity of malaria transmission is often associated with the presence of hotspots consisting of localized clusters of higher transmission intensity. This study aims to identify micro-geographic hotspots of malaria transmission in an agricultural setting using a multidisciplinary approach.

**Methods:**

Two cross-sectional surveys were conducted at the end of the dry season and at the peak of the rainy season in rural and urban sites in Bouna, northeastern Côte d'Ivoire. A total of 296 individuals from 148 farming households were randomly selected and sociological, geographical, entomological, and clinical data as well as blood samples were collected during each visit. Parasitological data and *Anopheles* exposure (measured using entomological and immunological methods) were compared with demographic, agricultural, and geographic data to identify drivers of malaria transmission. Heat maps combining these data were used to identify households with ongoing malaria transmission throughout the year.

**Results:**

In rural areas, *Plasmodium* prevalence was consistent between the dry and the rainy seasons, with roughly half of the population infected. In urban areas, malaria transmission indicators were lower, with a parasite prevalence of less than 20%, which remained comparable between the dry and the rainy season. The presence of irrigated crops and proximity to wetlands were associated with increased *Anopheles* exposure. By mapping *Plasmodium* infection and *Anopheles* exposure, two different types of hotspots of malaria transmission were identified: micro-geographical scale and local scale hotspots.

**Conclusions:**

The presence of wetlands in urban areas and irrigated agriculture in rural areas resulted in heterogeneity in malaria transmission on a micro-geographical scale. These specific households present particular risk of malaria transmission and could fuel malaria transmission in surrounding households. The identification of micro-geographical areas using heat maps combining several epidemiological parameters can help to identify hotspots of malaria transmission. The implementation of malaria control measures, such as seasonal chemoprophylaxis or vector control, in these areas could help to reduce the incidence of malaria and facilitate its elimination.

## Background

Substantial reductions in the global burden of malaria were noted during the past two decades in response to improvement of control measures with the mass distribution of long-lasting insecticidal nets (LLIN), access to diagnosis with rapid diagnostic tests (RDT) and prompt treatment with artemisinin-based combination therapy (ACT). Over the last few years, progress has stagnated with global malaria incidence and deaths remain stable with over 200 million malaria cases and more than 600,000 deaths in 2021 [1], confirming the need of new approaches to reach elimination goal. Since the COVID pandemic, upsurges have been noted in many sub-Saharan countries, including Côte d’Ivoire, where *Plasmodium falciparum* malaria is the leading cause of mortality among children [2].

*Plasmodium falciparum* transmission is driven by ecological factors including those that favoured *Anopheles* breeding sites and thus *Anopheles* populations. The presence of wetland as well the type of agricultural crops and practices, particularly irrigated cultures can create suitable breeding habitats for *Anopheles* mosquitoes, because mosquitoes require stagnant water for their larval habitat. A study in Kenya showed that irrigated agriculture was associated with a higher malaria transmission rate, likely due to increased mosquito breeding in the irrigation canals [3]. Similarly, a study in Mali reported that agricultural practices, particularly irrigation, were a major risk factor for malaria transmission in the region [4]. The watering of the agricultural areas during the dry season can also promote local malaria transmission resulting in a risk of malaria transmission throughout the year [5, 6]. Thus, the presence of wetlands nearby households might also be an ecological factor that influence spatial and temporal malaria transmission dynamic resulting in a heterogeneity of transmission. The households located nearby wetlands or irrigated crops might be particularly at risk and might clustered and represented hotspots where malaria is sustained throughout the year. During the dry season, the hotspot maintains transmission within the geographic area while during the rainy season, it fuels the transmission in surrounding areas [7]. As a result, understanding the link between land use, crop practices, and malaria transmission is crucial for designing effective control strategies and reducing the disease burden in endemic regions. Although complex, identifying malaria transmission hotspots is important for malaria elimination efforts. Thus, the identification of malaria hotspots that are stable in space and time might help to implemented targeted interventions to these specific areas, such as mass drug administration (MDA) or seasonal malaria chemoprevention (SMC).

In recent years, serological markers have been developed to assess individual level of exposure of human population to *Anopheles* bites. More specifically, the IgG response to the *Anopheles gambiae* gSG6-P1 salivary peptide has been identified as a proxy of exposure to *Anopheles* bites [8]. Studies in malaria endemic countries have shown that the use of this serological indicator is suitable to identify heterogeneity of exposure to *Anopheles* bites [9, 10], to evaluate the efficacy of vector control interventions and to identifying individuals at higher risk of malaria [11, 12].

This present study aimed to assess the role of wetlands and agricultural practices in malaria transmission in agricultural households in rural and urban settings in Bouna, Côte d’Ivoire, using several epidemiological indicators of malaria transmission. Mapping together these epidemiological parameters helped us to identify households the most at risk of malaria transmission in the different settings.

## Methods

### Study sites

**Fig. 1:**
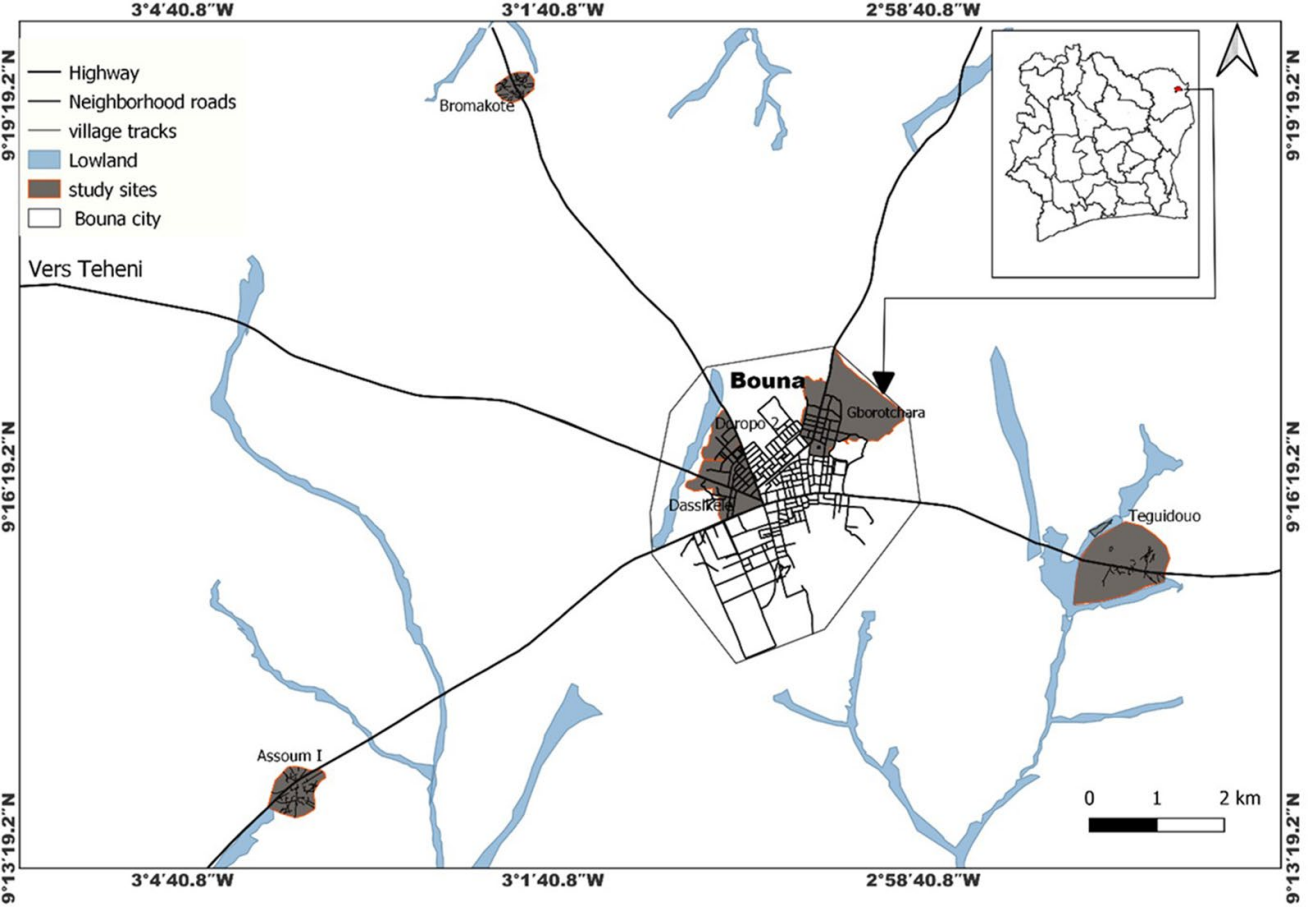
Map of Bouna area, showing the location of the six study sites

The study was conducted in the city of Bouna located in the North-East of Côte d'Ivoire (9°16' N,3°00' W) at the northern border to Burkina Faso and Ghana. The climate is tropical with two seasons including a dry season which runs from November through May and a rainy season from June to October. The annual rainfall recorded in 2020 was estimated at 847.4 mm with an average temperature around 26.7 °C. The study was carried out in 6 sites: 3 villages in rural setting (Assoum1, Téguiduo and Bromakoté) and 3 neighbourhoods in urban setting (Doropo2, Dassikeledougou and Gborotchara) (Fig. 1).

Excepted Gborotchara and Bromakoté, all these study sites are characterized by the presence of large areas of wetlands, some of which are used for irrigated rice cultivation and market gardening. The presence of domestic animals, such as cats and dogs, are quite remarkable as well as the breeding practices of cattle, goats, and poultry. The duration of the malaria transmission season is perennial with a peak in the rainy season. The most important malaria vector in this area is *An. gambiae sensu* stricto (*s.s.*) and *Anopheles coluzzii* [13, 14].

### Study design, procedure, and sample collection

Two cross-sectional surveys were carried out at the end of dry season (Mai 2021) and at the peak of the rainy season (August 2021), respectively. One hundred and forty-eight (148) agricultural worker households were randomly selected from the 2020 malaria registry from the city's general hospital. The malaria registry refers to a notebook used to collect and record information on malaria cases and related data (demographic). Three neighbourhoods and the 3 rural villages of Bouna with the highest malaria prevalence were selected. In each selected site, thirty households per site were randomly selected according to the inclusion criteria: households belonged to a selected site, households included one child who had been infected with malaria in the previous year and treated at the general hospital, and parents had an agricultural activity. Two people (at least one child) from each household were included. For households that were absent or whose members refused to participate, the nearest household was selected and included with their consent, resulting in a total of 296 participants.

Each household received a long-lasting insecticidal nets (LLINs) at the time of inclusion in late May 2021 during LLINs mass campaign distribution by the national malaria control program (NMCP). At each visit, sociological, geographical, entomological, and clinical data were collected. Participant’s axillary temperature was measured, and thick films and blood smears were performed to determine parasite density and *Plasmodium* species. Thick blood smears were fixed and stained with 10% Giemsa and read at double blind by certified microscopists. Asexual parasite densities were counted against 200 microscope fields white blood cells assuming 8,000 white blood cells per microlitre. A blood smear was considered negative if no parasites were observed. For immunological assays, dried blood spots (DBS) were collected at the fingertips on Whatman 3 MM filter, air-dried and stored in plastic bags at + 4 °C until immunological analysis. A household was defined as infected when at least one individual from this household was infected with *Plasmodium* (positive smear and *Plasmodium* density > 1 parasite/µL) during the survey.

### Mosquito collection

Adult mosquitoes were collected by human landing catches (HLC) in June and August in two reference households in each of the 6 sites. In each reference household, two catching points, one indoor and one outdoor were used to collect mosquitoes by landing catches on adult volunteers from 6.00 pm. to 6.00 am. Adult mosquito catchers gave prior informed consent, were vaccinated against yellow fever and were treated for malaria according to the national treatment guidelines. The mosquitoes were hourly collected, and carefully stored in closed hemolysis tubes with cotton and kept in plastic bag by time slot and by catching point. The human biting rate (HBR) of *An. gambiae* was calculated as the average number of mosquitoes collected per person per night (bites/human/night, BHN). The mosquitoes from HLC methods were identified morphologically at the species level. This identification was made according to the determination key of Gillies and De Meillon [15].

### Evaluation of the use of LLINs and inventory of agricultural practices

The use and physical integrity of the LLINs was recorded by the administration of questionnaires in all study households. Community health workers verified bednet installation after distribution of the news LLINs during the mass campaign. The questionnaires also recorded data on agricultural practices: nature of culture and type of irrigation practiced by household members.

### Measurement of human IgG level to Anopheles gSG6-P1 salivary peptide

Human IgG level against the gSG6-P1 salivary antigen of *An. gambiae* was measured by enzyme-linked immunosorbent assay (ELISA). Standardized dried blood spots (diameter: 1 cm) of each participant at two time points were eluted each in 300 µL phosphate buffered saline containing 0.1% Tween 20 (0.1% PBST) at 4 °C for 24 h. Subsequently, 96-well Maxisorp micro-assay plates (Nunc, Roskilde, Denmark) were coated with 100 µl of the gSG6-P1 salivary peptide (20 µg/mL in PBS) for 2 h and 30 min at 37 °C. Plates were blocked for 1 h with 200 μL of protein-free blocking buffer, pH7.4 (Thermo scientific, Rockford, USA). After washing, individual eluate was incubated in duplicate wells at 4 °C overnight wells at 1/40 dilution in 0.1% PBST. Mouse biotinylated anti-human IgG antibody (BD Pharmingen, San Diego, CA) was added at a 1:2000 dilution in 1%-PBST (1 h and 30 min at 37 °C), and peroxidase-conjugated streptavidin (Amersham, Les Ulis, France) was then added (1/15,000; 1 h at 37 °C). Colorimetric development was carried out using ABTS (2.2′-azino-bis (3 ethylbenzthiazoline 6-sulfonic acid) diammonium; Roche Diagnostics, Lyon, France) in 50 mM citrate buffer (pH 4) containing 4% H_2_O_2_ and the Optical Density (OD) was measured 2 h later at 405 nm. Individual results (ΔOD) are expressed as: ΔOD = ODx-ODn where ODx represents the mean of individual OD value in both wells containing the salivary antigen and ODn the individual OD value for each eluate without gSG6-P1 antigen.

### Maps

The geographic coordinates of the households were recorded using Global Positioning System 60 (GPS) units (GARMIN, Gsmap, 64sx) with an accuracy of 5 m. The houses selected for the entomological study and the main breeding sites of vectors were georeferenced. The data on *Plasmodium* density, IgG level to the gSG6-P1 salivary peptide and the *Anopheles* HBR were spatially linked to the GPS data of each household. Once these results were entered into the attribute table of the QGIS software, different intervals were defined automatically for mapping purposes. Finally, the HLC site location were plotted in the map. An interpolation using the inverse distance weighting method was carried out with pixels of equal columns and rows of 254 for a better rendering of the capture points. The maps were analysed on the basis of the spatial and temporal dynamics of epidemiological parameters related to malaria transmission (IgG level to the gSG6-P1 peptide and *Plasmodium* prevalence and density). Hotspots represent groups of households with high values of malaria transmission parameters in the dry and in the rainy season. Hotspots were categorized into two categories according to their spatial scale: micro-geographical hotspots refer to small spatial areas (within a radius of 100 m), involving clusters of few houses, while local hotspots encompass a larger spatial extent, from 0.1 to 1 km, including an entire neighbourhood or village.

### Measuring household distance to the wetland

The measurement of the distance between household and the nearest wetland (edge) was conducted using direct measurements with a GPS. During this phase, the GPS automatically recorded the traveled path as a line and a succession of points. Participants were divided into 2 groups according to the distance between households and the nearest wetland. The arbitrary threshold (= 400 m) was determined on the basis of the value of the median (= 423 m) and mean (= 390 m) of the distance between households and the wetland.

### Statistical analysis

A database was developed using Microsoft Excel software and then the data was analysed with Graph Pad Prism 9 (Graph Pad Software, San Diego, USA) and R software (Version 1.2.5033; R Core Team, Vienna, Austria). After checking that the data did not follow a Gaussian distribution (normal distribution), the non-parametric Mann–Whitney U-test was used to compare the IgG levels between two independent groups, and the non-parametric Kruskal–Wallis test was used for comparison of the IgG levels between more than two groups and the Dunn’s post-hoc test was also performed for two-by-two comparisons in more than two groups. The paired Wilcoxon test was used to compare IgG response levels between the two time points in each setting or site. The Spearman’s rank correlation test was used to analyse the correlation between the distance of the household to the nearest wetland with the ΔOD of tested sera and with the parasitic density (log10). Logistic regression was performed to assess the relationship between the distance of the household to the nearest wetland and the prevalence of *Plasmodium* infection. The HBR was compared between sites by using the prop.test testing the null hypothesis that the proportions in several groups are the same. All differences were considered significant at p < 0.05.

## Results

### Study population characteristics, parasitological and entomological data according to setting and sites

Characteristics of the study population (gender ratio and age), parasitological (prevalence and geometric mean of *P. falciparum* density) and entomological data (*Anopheles* Human Biting Rate) are presented according to study sites and season in Table 1.

**Table 1:** Characterization of populations, prevalence, parasty density of *Plasmodium falciparum* and *Anopheles* human bite rate

		n	Mean age	Gender ratio	Dry season			Rainy season		
					Parasite prevalence (%)	Parasite density (95%CI)	*Anopheles* HBR (bpn)	Parasite prevalence (%)	Parasite density (95%CI)	*Anopheles* HBR (bpn)
			(95%CI)							
Rural setting	Assoum1	46	10.81	1.55 (28/18)	18 (39.13)	396.93 (125–670)	64.12	20 (43.47)	7675.30 (7403–7947)	109
			(6.54–15.09)							
	Bromakote	54	9.52	0.86 (25/29)	30 (55.55)	3232 (1504–4959)	27.25	30 (55.55)	4942.93 (3215–6670)	58
			(6.69–12.36)							
	Teguiduo	52	7.18	0.73 (22/30)	30 (57.69)	3193.17 (425–5961)	66.37	32 (61.53)	2858.65	67
			(4.98–9.38)						(90–5927)	
Subtotal		152		0.97 (75/77)	78 (51.31)	2360.73	52.58	82 (53.95)	5056.79 (2519–7594)	78.3
			9.11			(1217–3504)	(IC = 0–125)			
			(7.30–10.92)							
Urban setting	Gborotchara	44	10.64	1.20 (24/20)	7 (15.90)	472.70 (0–1278)	2.75	9 (20.45)	372 (195–549)	55
			(6.85–14.44)							
	Dassikeledougou	46	10.65	0.77 (20/26)	12 (26.08)	1495.02 (76–2914)	4.12	7 (15.21)	269.56 (0–1688)	54.25
			(6.77–14.54)							
	Doropo2	56	7.67	1.77 (28/26)	9 (16.66)	470.24 (37–903)	77.5	11 (19.64)	1363.33 (930–1796)	115.25
			(4.66–10.70)							
Subtotal		146		1.13 (82/72)	28 (19.17)	798.35	28.12	27 (18.49)	711.02 (144–1277)	58.42
			9.53			(256–1340)	(IC = 1–113)			(IC = 0–185)
			(7.50–11.68)							
TOTAL	Mean	296	9.32	0.98 (147/149)	106 (35.81)	1600.65	40.35	109 (36.82)	2942	68.37
			(7.96–10.68)			(952–2249)			(1590–4295)	

In total, 296 participants aged from 0 to 80 years (mean age = 9.32; 95% confidence interval (95% CI 7.96–10.68) were included in the study. Mean age of the participants was similar between study sites (p = 0.22).

In rural setting, the parasite prevalence was similar between the dry and the rainy season (p = 0.646) with an average of half of the population presented an infection to *P. falciparum*. According to village, in dry season it ranged from 39,1% (Assoum1) to 57,7% (Téguiduo) and from 43,5% (Assoum1) to 61,5% (Téguiduo) in rainy season. The malaria prevalence was not significantly different between the 3 rural sites in dry (p = 0.137) and rainy season (p = 0.193) and the prevalence was similar in each site between dry and rainy season. Globally, geometric mean parasite density was significantly different between the dry and the rainy season (p = 0.019). It was different between the 3 rural sites only during the dry season (p = 0.036) and two-by-two comparison using Dunn’s test indicated a statistical difference between Assoum1 and the other sites. The geometric parasite means in Assoum1 significantly increased between the dry and the rainy season (p < 0.001) while it was not significantly different in Bromakote (p = 0.928) and Téguiduo (p = 0.438). A high mean level of *Anopheles* bites was recorded in rural setting in the dry season (52.6 bites per human per night, BHN) and in the rainy season (78.3 BHN). The aggressivity of *An. gambiae* was higher in Assoum1 and Téguiduo in the dry season with about 65 BHN while 27 BHN was recorded in Bromakote. During the rainy season, the HBR significantly increased (double) to 109,5 BHN in Assoum (p < 0.001), and to 58 BHN in Bromakote (p = 0.001), while it stayed similar in Téguiduo.

In urban setting, epidemiological indicators of malaria transmission were lower, with a mean of parasite prevalence less than 20% in the dry and rainy season. In dry season, the highest *Plasmodium* prevalence was observed in Dassikeledougou (26.1%) while the sites of Gborotchara and Doropo presented a prevalence of 16% but the difference between the 3 urban sites was not significant (p = 0.384). In the rainy season, the prevalence was also similar between the 3 urban sites (p = 0.758) with Gborotchara (20.45%) and Doropo (19.6%) presented the highest rate. The prevalence was similar in each urban site between the dry and the rainy season. Geometric parasite mean was similar between the 3 urban sites during the dry (p = 0.842) and the rainy season (p = 0.705). No significant changes in the geometric parasite mean were noted between the dry and the rainy season in the urban setting and in each site. The HBR of *An. gambiae* was also globally lower than in rural setting with approximately 5 BHN recorded in Gborotchara and Dassikeledougou in the dry season and about 55 BHN in rainy season. Doropo presented the highest *An. gambiae* HBR both in the dry (77.5 BHN) and in the rainy (115.25 BHN) season. *Anopheles* aggressiveness significantly increased in all urban sites between the two seasons (p < 0.01).

### IgG response to gSG6-P1 peptide according to setting and season

**Fig. 2:**
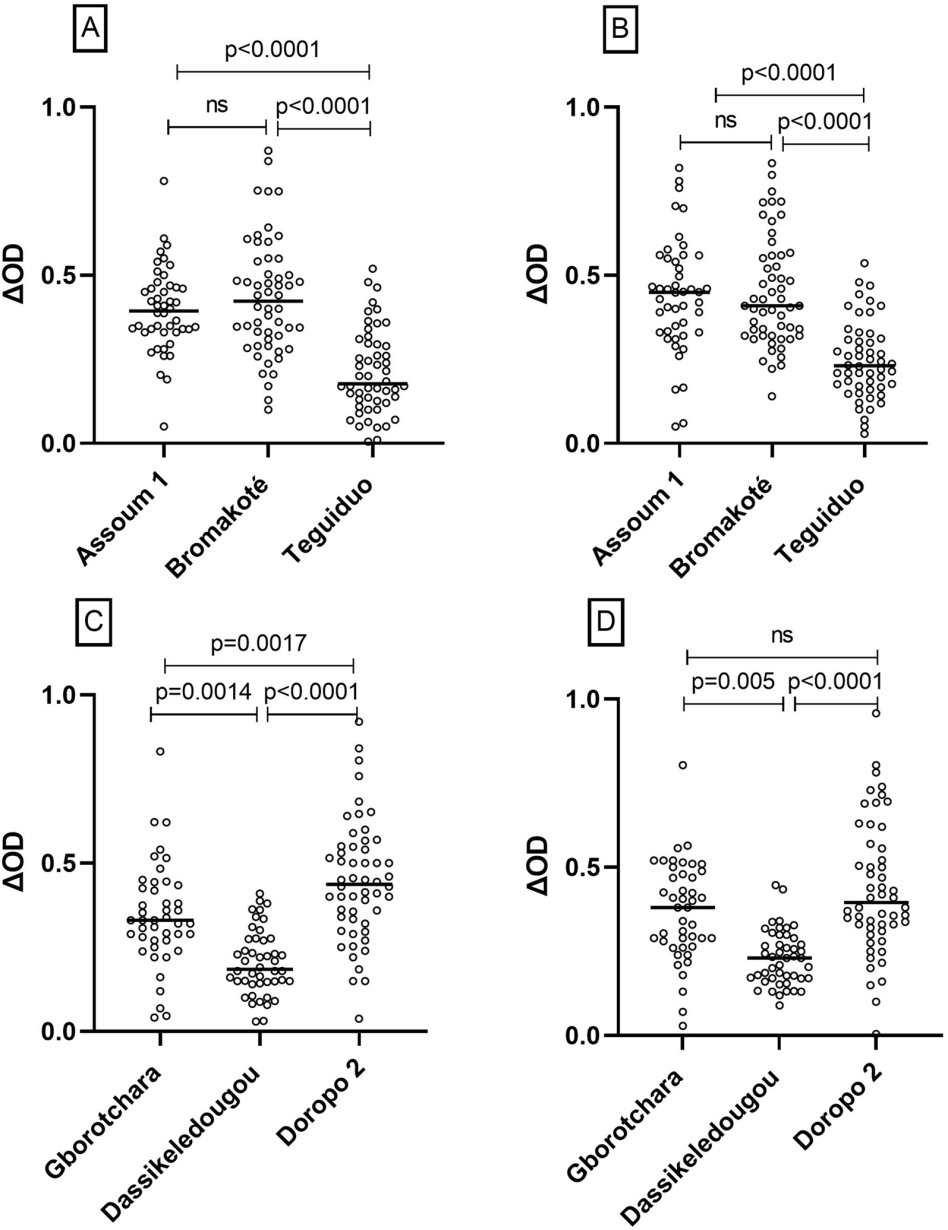
Immunoglobulin G (IgG) responses to gSG6-P1 in each site according to setting and season. The statistical difference in IgG response levels between different parameter categories was indicated by P values, estimated using the Kruskal–Wallis test and Dunn's multiple comparison test. **A** = Rural sites in dry season; **B** = Rural settings in rainy season; **C** = Urban sites in dry season; **D** = Urban settings in rainy season

The specific IgG response level to the *Anopheles* gSG6-P1 salivary peptide represents an individual proxy of the level of exposure to *Anopheles* bites and was assessed in participants in each site during the dry and the rainy season. Participants presented a wide range in ∆OD_gSG6-P1_ within and between the different sites, both in the dry and the rainy season (Fig. 2).

In rural setting, the IgG responses were significantly different between the 3 villages both in dry and rainy season (p < 0.0001) (Fig. 2A, B). During the two seasons, there was no significant difference in IgG median level between the site of Assoum1 and Bromakoté. The IgG median level in Téguiduo was significantly lower to Assoum and Bromakoté both in the dry and the rainy season (p < 0.0001, Dunn’s test two-by-two comparison). A significant increase of IgG levels between the dry and the rainy season was recorded only in Téguiduo site (p = 0.037, Wilcoxon test).

In urban setting, the IgG responses were significantly different between the 3 neighbourhoods both in the dry and the rainy season (p < 0.0001) (Fig. 2C, D). Dassikeledougou presented a significantly lower IgG level to gSG6-P1 antigen compared to Doropo2 and Gborotchara both in the dry and the rainy season (p < 0.0001 and p < 0.005, respectively, Dunn’s test two-by-two comparison). Gborotchara showed a significant lower IgG level to gSG6-P1 antigen compared to Doropo2 in the dry season (p = 0.0017) while a similar median level of IgG response was recorded in rainy season (p = 0.111, Mann Whitney Test).

### IgG response to gSG6-P1 peptide according to agricultural practices (irrigated/non-irrigated)

**Fig. 3:**
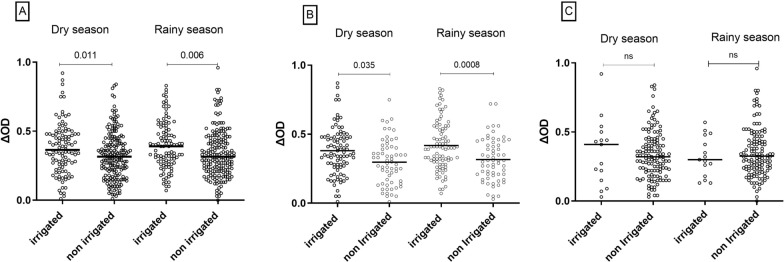
Immunoglobulin G (IgG) response level to gSG6-P1 according to agricultural practices. Statistical difference of the level of IgG responses between different setting categories was indicated by *P* values, estimated using the Kruskal–Wallis test and Mann Whitney test. **A** = Total population; **B** = Rural setting; **C** = Urban setting

IgG responses to the *Anopheles* gSG6-P1 salivary peptide were compared according to the agricultural practices of participants from each setting and site according to season (Fig. 3).

The cultures practiced by the population have been categorized into two groups: irrigated crops and non-irrigated crops. Irrigated crops were composed of market gardening, rice, and cereals (millet, corn), while non-irrigated crops included shea, cashew, coffee, cocoa, and yam.

Considering the 2 settings together, IgG median level responses to gSG6-P1 peptide was significantly higher in participants practicing irrigated crops compared to those producing non-irrigated crops, both during the dry (p = 0.011) and rainy season (p = 0.006) (Fig. 3A). The same observation was noted in the rural setting (p = 0.035 in the dry season and p = 0.0008 in the rainy season) (Fig. 3B), while a similar IgG median level was noted in the urban context between participants practicing irrigated or not irrigated crops in the dry and in the rainy season (Fig. 3C). In both settings, people practicing irrigated or non-irrigated crops presented similar level of anti-gSG6-P1 IgG level between the dry and the rainy season.

### IgG response to gSG6-P1 peptide according to distance between household and wetlands

**Fig. 4:**
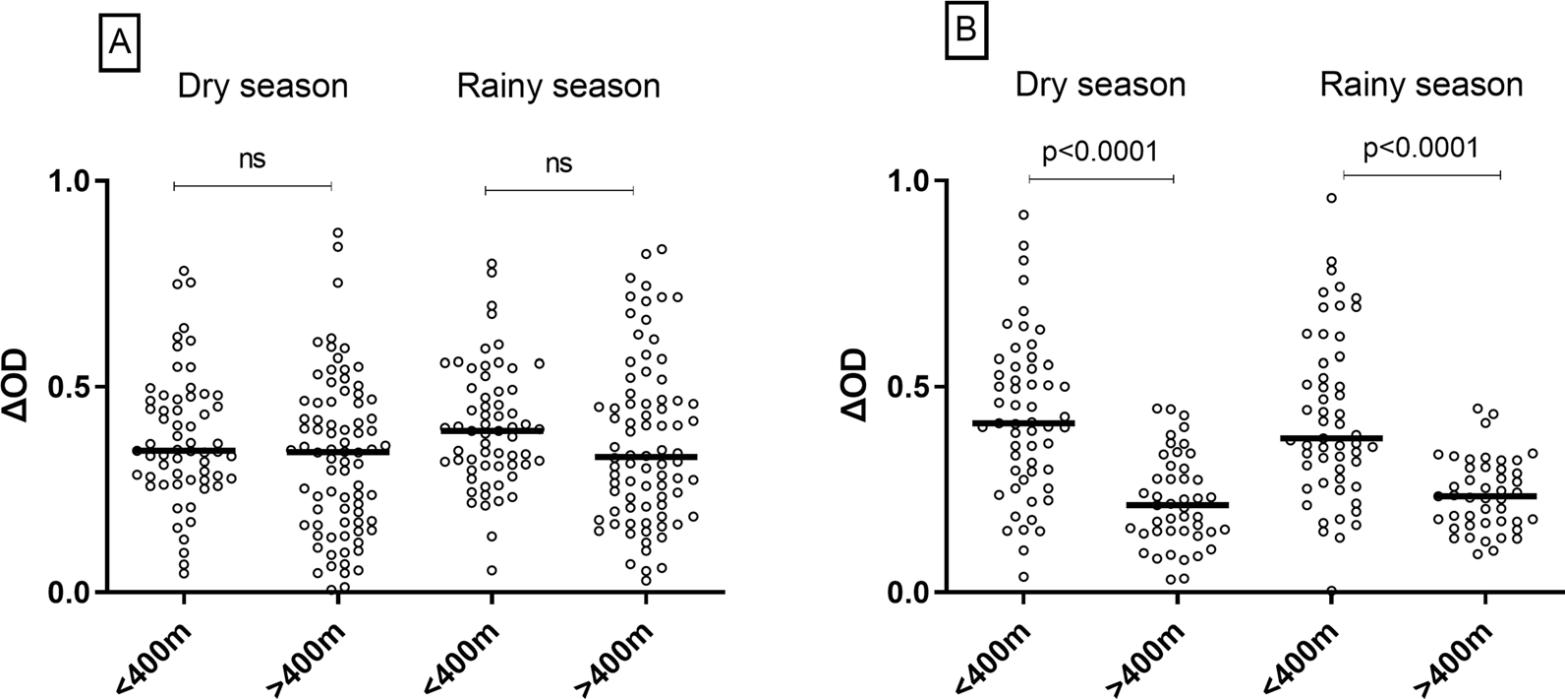
Immunoglobulin G (IgG) response level to gSG6-P1 according to distance from households to wetlands. Statistical difference of the level of IgG responses between different setting categories was indicated by *P* values, estimated using the Mann Whitney test. **A** = Rural setting; **B** = Urban setting

The distance of each household from the nearest wetland was measured and households were then categorized in two groups according to the distance: households located less than 400 m from the wetland, or more than > 400 m (Fig. 4).

In rural setting, no significant difference in anti-gSG6-P1 IgG level was noted between the two groups of households during the dry (p = 0.143) and the rainy season (p = 0.096) (Fig. 4A). Only the households located more than 400 m to the wetland presented a significantly different IgG level in the rainy season compared to the dry season (p = 0.044). In urban setting, households close to the wetland presented a significantly higher level of IgG response compared to the households far from the wetland both in the dry (p < 0.0001) and the rainy season (p < 0.0001) (Fig. 4B). No significant difference was noted for each group of households between the dry and the rainy season.

### Association between malaria transmission and distance from households to wetlands

**Fig. 5:**
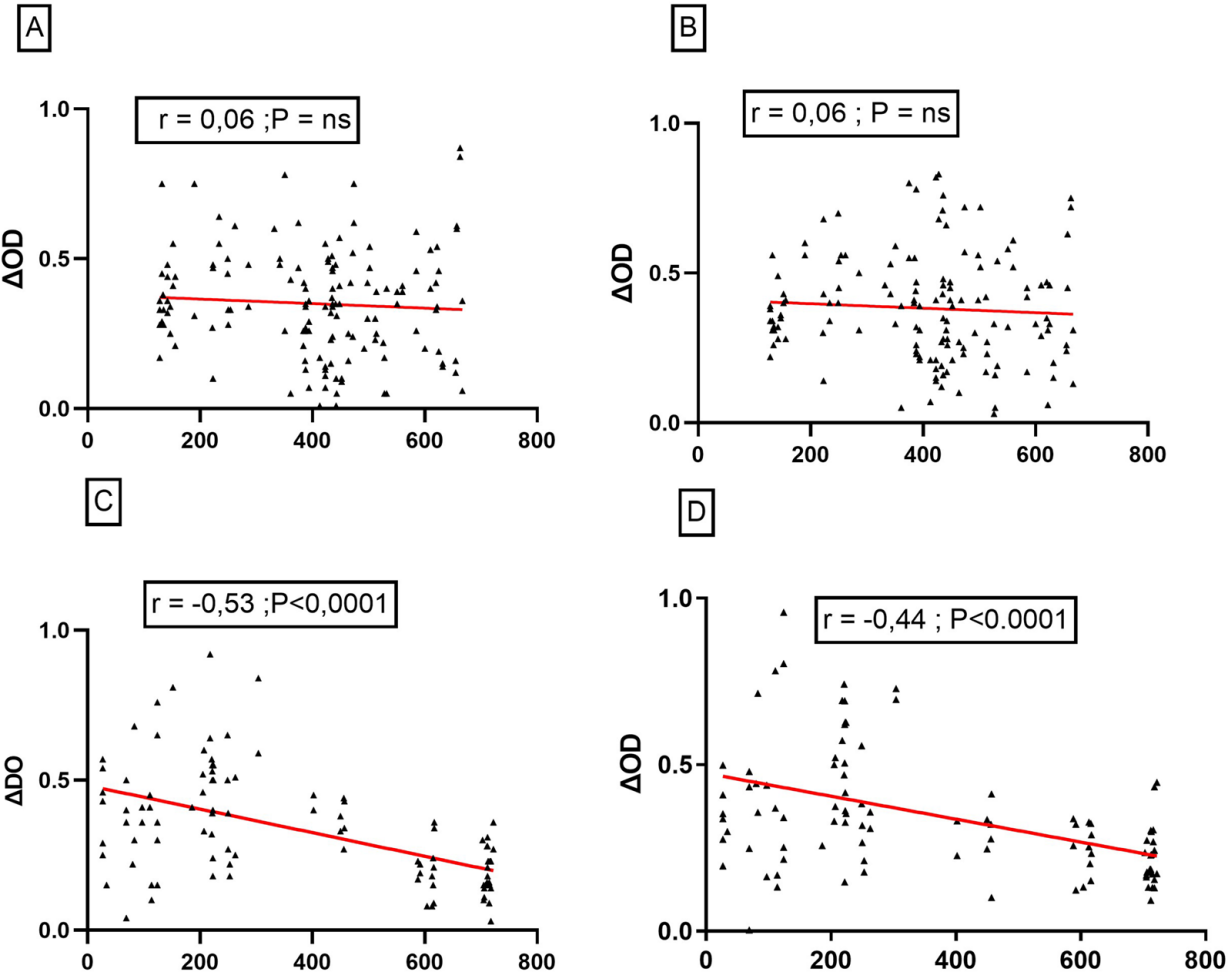
Correlation between Immunoglobulin G (IgG) response level to gSG6-P1 and the distance from households to wetlands. Scatter plot analysis of IgG responses to salivary antigens and distance from each households to the near shallows is presented. **A** = Rural setting in dry season; **B** = Rural setting in rainy season; **C** = Urban setting in dry season; **D** = Urban setting in rainy season

To decipher the role of wetlands in malaria transmission risk, we investigated the variation in anti-gSG6-P1 IgG level, *Plasmodium* prevalence and density according to the distance of the households to the wetlands. The correlation between the IgG levels specific to the salivary antigen and the distance to wetland was compared for each participant individually in the two settings separately using a Spearman's rank correlation test, and the corresponding *p-values* were determined (Fig. 5).

**Fig. 6:**
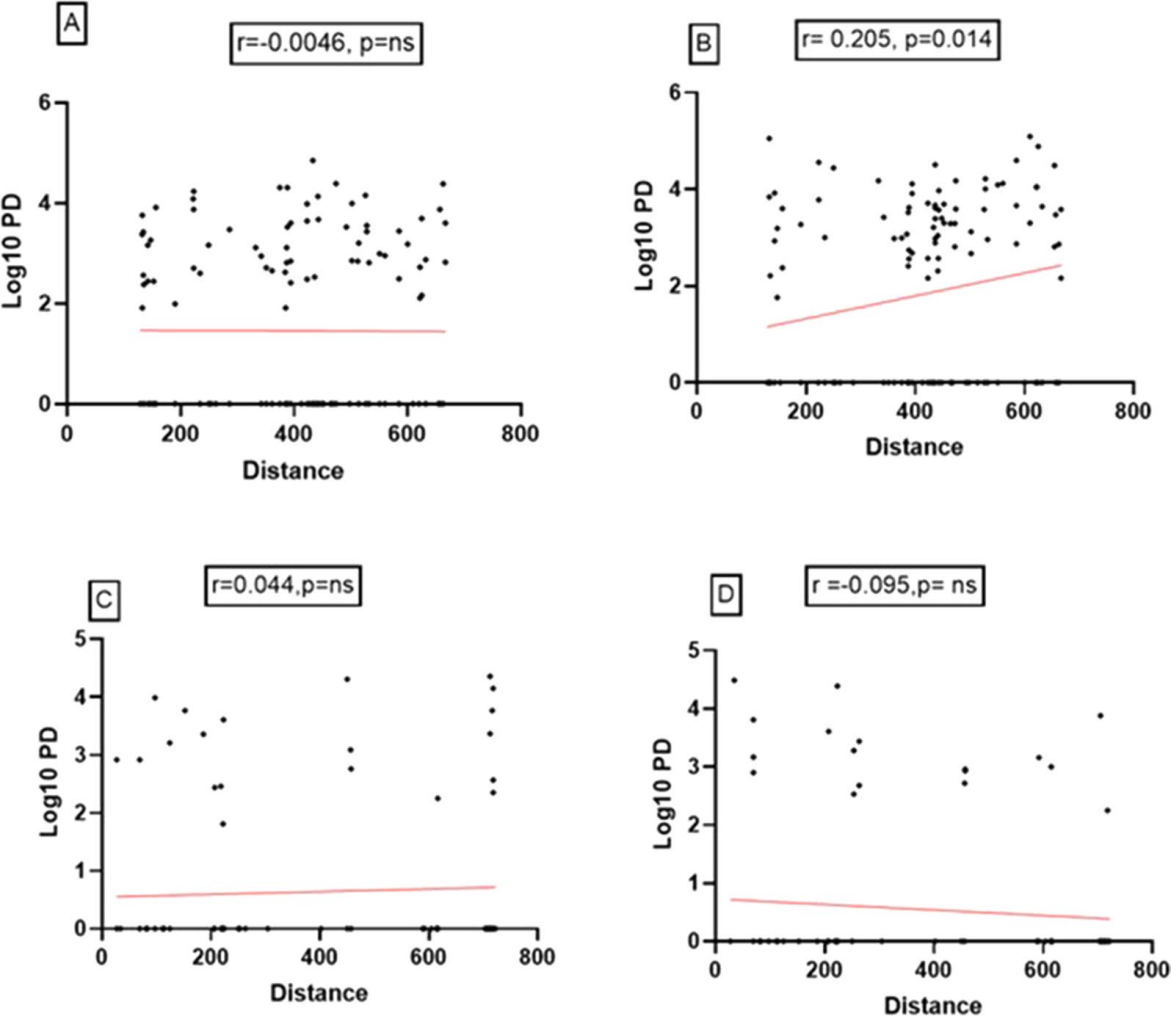
Correlation between parasite density (Log10) and the distance from households to wetlands. Scatter plot analysis of parasite density and distance from each households to the wetlands is presented. **A** = Rural setting in dry season; **B** = Rural setting in rainy season; **C** = Urban setting in dry season; **D** = Urban setting in rainy season

A moderate significant negative correlation between the anti-gSG6-P1 IgG level and the distance to wetland was found only in urban setting in the dry (r = −0.53, p < 0.0001) and in the rainy season (r = −0.44, p < 0.0001). No correlation was found in the rural setting between the IgG level and the distance to wetland. Similar statistical analyses were performed to assess the correlation between *Plasmodium* density (log10) and the distance to wetland (Fig. 6).

A significant positive association between parasite density and distance from wetlands to household was showed in rainy season in rural setting (r = 0.23, p = 0.007). We also observed a positive association between *Plasmodium* prevalence and the distance to wetland in rural setting in rainy season (p = 0.033).

### Season maps of risk of exposure to *An. gambiae* bites

**Fig. 7:**
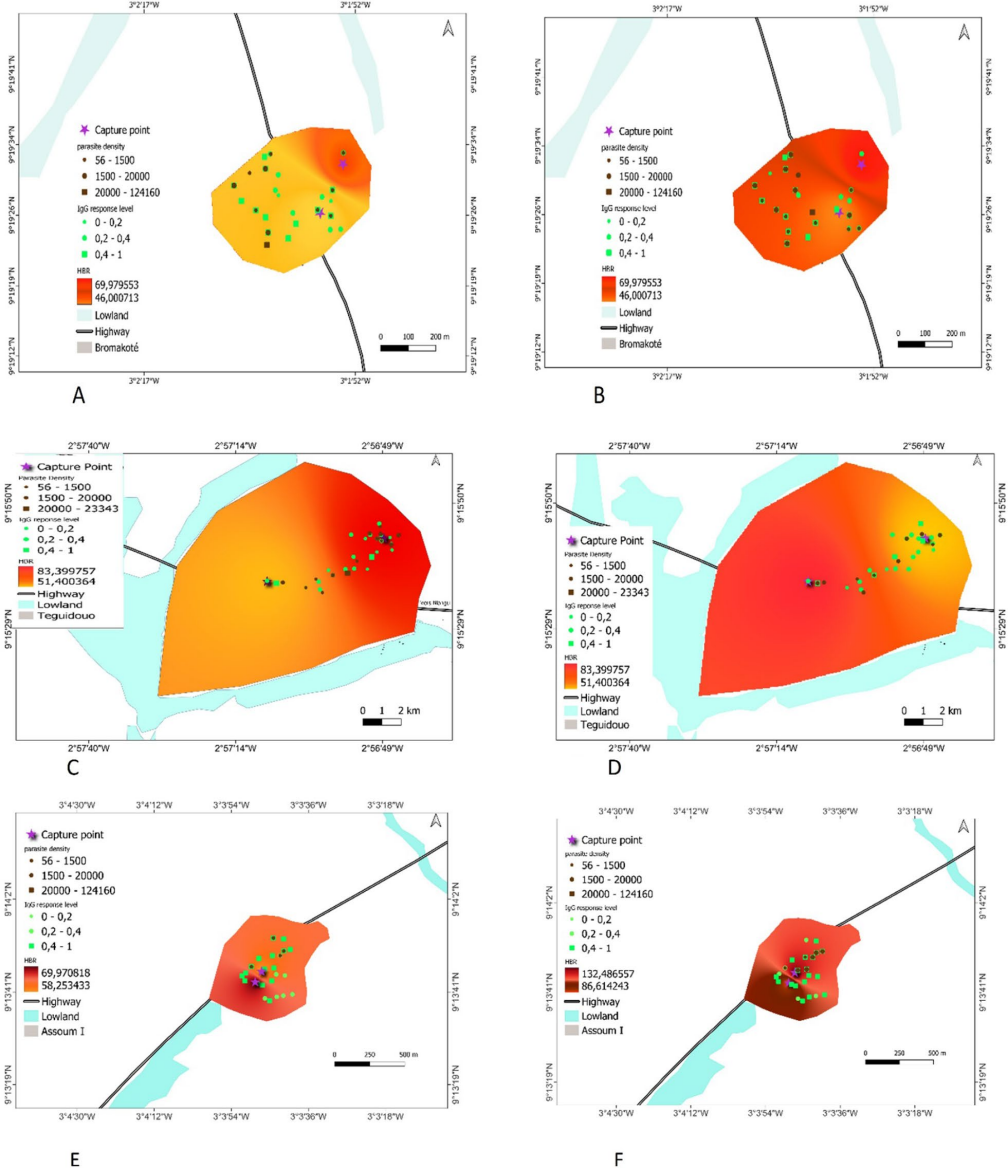

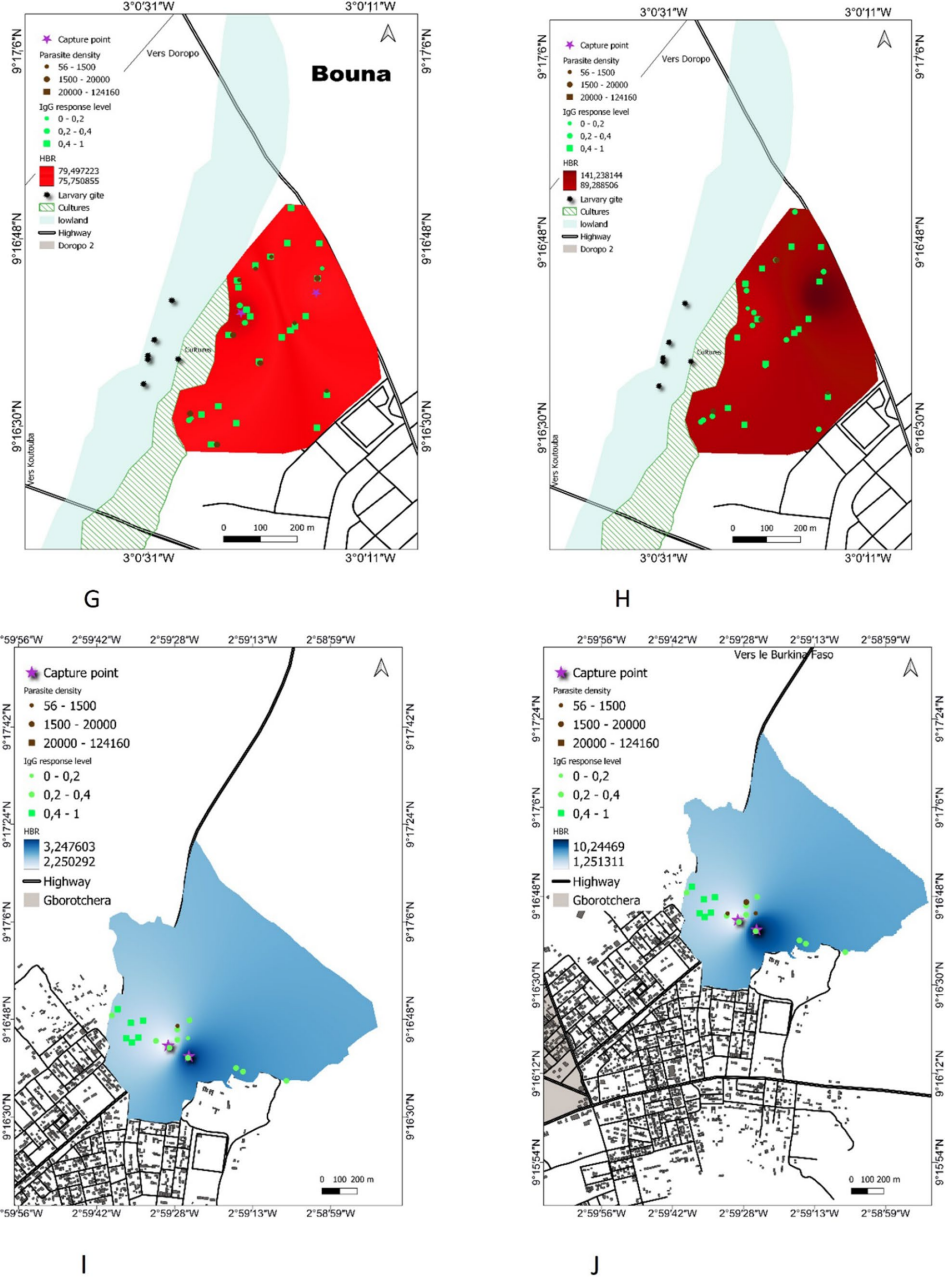

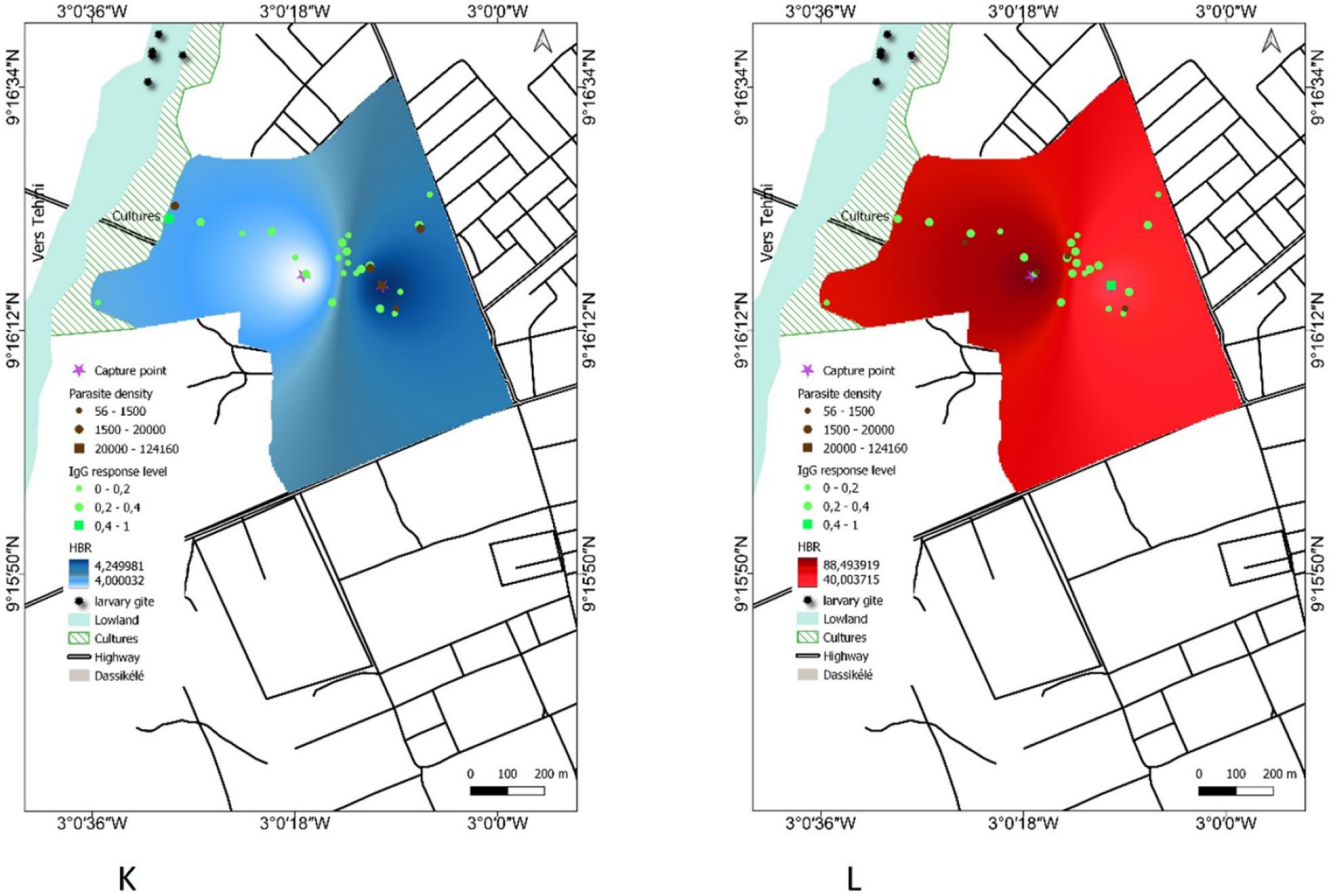
Maps of malaria exposure and transmission for each site in the dry and the rainy season. Bromakoté (**A**, **B**), Téguiduo (**C**, **D**), Assoum1 (**E**, **F**), Doropo2 (**G**, **H**), Gborotchara (**I**, **J**), Dassikélédougou (**K**, **L**). Parasite density and IgG level to the gSG6-P1 antigen are represented by bright green or black color, respectively. Circles represent low to medium intensities and the highest values is indicated by square. The households where entomological surveys have been conducted are indicated by purple cross

Maps were generating by combining indicators of malaria transmission (anti-gSG6-P1 IgG response, HBR and mean of parasite density in each study site during the dry and the rainy season (Fig. 7).

We analyzed the maps based on the spatial and temporal dynamics of exposure to *Anopheles* bites (individual level), and *Plasmodium* prevalence and density. Maps indicated variation in the distribution of the IgG levels to the gSG6-P1 peptide and the levels of parasitaemia across space and season according to the sites. In Bromakoté, there was a high homogeneous risk of exposure to *Anopheles* bites and infected households were scattered throughout the village both during the dry and the rainy season (Fig. 7A, B), as observed in the urban setting Doropo2 (Fig. 7G, H) and in the rural site of Téguiduo (Fig. 7C, D).

In some villages the highest level of malaria indicators were observed in the same households during the two seasons. For example, in Assoum most households were at a high risk of exposure to *Anopheles* bites, but infected households were clustered in a patch in the northern part of the village during the two seasons (Fig. 7E, F). In the urban setting, Gborotchara is a highly urbanized neighbourhood with no presence of wetland. The risk of exposure and infection was low during the two seasons, with the highest exposed households located in the western part of the site while the infected household were in a patch in the centre (Fig. 7I, J). In Dassikeledougou, although the wetland was not exploited, market gardening was practiced on its edge. Exposure to *Anopheles* bites increased between the two seasons and was homogeneous within the site, while the few infected households were in different place according to the seasons (Fig. 7K, L).

## Discussion

This work aims to explore the transmission of malaria in an agricultural context and to specifically investigate the impact of the presence of wetland areas and agricultural practices on the heterogeneity of malaria transmission in rural and semi-urban sites. This study aims to gain a deeper understanding of the environmental factors that contribute to the spread of malaria in the health district of Bouna, Côte d’Ivoire. In the present study, different epidemiological parameters were investigated, namely *P. falciparum* prevalence and density, *Anopheles* aggressiveness and the IgG response to the gSG6-P1 salivary peptide that represents a proxy of exposure to *Anopheles* bites.

Exposure to *Anopheles* bites was thus investigated via two complementary methods giving different level of information about the exposure to *Anopheles* bites. First, the entomological indicator using the human landing catches (HLC) that indicates the mean number of bites that an individual may receive per night, appreciates as an approximate proxy the level of exposure to *Anopheles* at site scale. Second, a serological biomarker of exposure based on the quantification of the IgG response specific to the *An. gambiae* gSG6-P1 salivary peptide was used to evaluate the human–*Anopheles* contact at the individual level. This tool integrates the individual risk factors of being bitten and provides for each participant a proxy of the individual level of exposure to *Anopheles* bites [8]. It is more appropriate for reflecting the inter-individual heterogeneity of exposure (attractiveness to mosquito, use of personal protection as bed net, for example) in natural setting [9]. Numerous studies have evidenced the *Anopheles* gSG6-P1 salivary peptide represents a reliable and complementary tool to entomological methods for assessing spatial and temporal heterogeneity of exposure to *Anopheles* bites at the individual level [16]. In the present study, malaria transmission was heterogeneous between the 6 studied sites, with a prevalence of *Plasmodium* infection ranging from 15% to nearly 60% in dry season and in rainy season according to site. Malaria transmission was higher in rural sites where almost half of the population was infected compared to urban neighborhoods. Exposure to *Anopheles* bites was also heterogeneous in Bouna during the study, with various level of *Anopheles* aggressiveness according to site. Heterogeneity between and within sites is also reflected by the heterogeneity in the individuals’ anti-gSG6-P1 levels.

*Plasmodium* prevalence was similar in each site between the dry and the rainy season both in the rural and the urban setting, indicating the malaria transmission is perennial throughout the year in the city of Bouna and in its surroundings. It may also suggest that persistent asymptomatic infections occurred in the urban setting, where only few *Anopheles* were caught during the dry season. In the rural setting, entomological studies reported the presence of a high density of *Anopheles* mosquitoes throughout the year with a mean of 52 bpn and 78 bpn recorded during the dry season and the rainy season, respectively. The presence of wetlands and the agricultural practices (irrigated rice culture and market gardening) could create potential breeding sites for *Anopheles* mosquitoes during the dry season that may sustained exposure to *Anopheles* and thus malaria transmission throughout the year. In the rainy season, new temporary *Anopheles* breeding sites could be created with the water rains and came to be added to the permanent breeding sites that wetland could represent. Interestingly, Bromakote and Gborotchara, the two sites without the presence of wetlands, had the lowest density of *Anopheles* for each setting, both in the dry and the rainy season.

To decipher the role of agricultural environment in the risk of malaria exposure, the variation of the IgG level to the gSG6-P1 salivary peptide during each survey (dry and rainy season) and in each setting according to agricultural practices was first studied. The type of culture influenced the individual exposure to *Anopheles* bites. In rural setting, individuals that practiced irrigated culture (rice, corn, market gardening) presented a significant higher exposure to *Anopheles* compared to individuals practicing non–irrigated cultures, both during the dry and the rainy season. Individuals can be either more exposed during the field activities or surrounding their households. In rural setting, irrigated crops or market gardening are commonly grown near households, and thus can increase the risk of being bitten [17]. This highlighted the role of irrigated crops in the sustainability of malaria risk transmission in providing breeding sites for *Anopheles* mosquitoes throughout the year. In urban neighborhoods, there is limited space between dwellings and cultural practices are restricted in peri-urban districts [18]. This is reflected by the low number of individuals who reported practicing irrigated culture in urban setting. Secondly, a significant negative correlation between the level of IgG level and the distance of the households of participants to the nearest wetland in urban setting was noticed. This evidenced that proximity to wetlands, which are often associated with agricultural activities, may increase the exposure to *Anopheles* mosquitoes. This is consistent with previous studies that have identified the role of agricultural activities and the presence of stagnant water in promoting malaria transmission [4, 19]. In rural areas, where no such association was found, the presence of particularly atypical larval habitats close to households, such as water tanks, abandoned containers or irrigated crops, may also represent breeding sites and thus offset the effect of wetlands [20, 21].

In this study, no association was observed between the distance to wetlands and the *Plasmodium* density and prevalence. A significant positive association was observed in rural setting in rainy season. Altogether, this means that individuals living near wetlands were more exposed to *Anopheles* but did not present a higher risk of malaria transmission. Such apparent paradox has already been described, namely the paddy paradox [17, 22].

Then, we mapped the different epidemiological indicators of malaria transmission on maps for the dry and the rainy season, with the aim to identifying households or groups of households at high risk of malaria transmission throughout the year. Two types of hotspots which differed according to their spatial scale were identified. The first type is represented by micro-geographical areas within a study site, which is composed of a group of households where the risk of exposure and infection was localized and persistent between seasons. In Assoum1 and Gborotchara, malaria hotspots were composed of a group of infected households concentrated in a patch, persisting during the two seasons and from which transmission spread during period of high transmission with surrounding households infected in rainy season. In other sites, Téguiduo, Doropo2 and Bromakoté, malaria transmission was homogeneous, and thus the entire site is at risk of malaria transmission thus representing a local hotspot.

The contrasting outcomes concerning malaria transmission pattern within irrigated areas highlight the significance of considering local context, environmental variations, and human behavior when assessing malaria risk. Targeted interventions that consider the specific factors of risk in different settings would be more effective in reducing malaria transmission and ultimately improving public health outcomes [23]. Also implement integrated vector control management strategies alongside irrigation practices could help to reduce malaria transmission in endemic areas. Therefore, effective management of wetlands by employing various methods such as environmental management (levelling land, proper drainage) and biological control will reduce the breeding sites and proliferation of *Anopheles* [24, 25]. A harmonization of the agricultural practice would be also essential to better control the proliferation and to manage the resistance to the insecticides related to the pressure of selection emanating from the anarchic use of the pesticides. Harmonization of irrigated agricultural practices involves farmers planting at the same time of malaria control effort. It can improve efficiency by reducing breeding sites, concentrating resources, promoting integration, sharing data, and involving communities.

In sites where the risk is high and homogeneous, such as Doropo2, Bromakoté, and Téguiduo, seasonal malaria chemoprophylaxis could be initiated before the start of the rainy season, with the aim reducing *Plasmodium* reservoir and preventing the spread of infection.

This study has several limitations. Firstly, due to logistical constraints, only 2 capture points in each site were used for the entomological studies. As a result, the aggressiveness of *Anopheles* was extrapolated from these 2 capture points, and the interpolation on the maps should be interpreted with caution. Nevertheless, measuring individual exposure using the serological approach overcomes this limitation. In addition, the identification and counting of potential *Anopheles* mosquito breeding sites close to households would have enabled them to be taken into account. A more complete characterization of the wetland, such as the measurement of standing water levels and the study of *Anopheles* larvae, would also have provided additional environmental characteristics to be tested, in addition to the type of agricultural crops. The intricate interplay between agricultural practices, notably rice crops, and malaria transmission underscores the need for a comprehensive understanding of factors influencing vector dynamics. Members of the *Anopheles gambiae* complex, particularly *Anopheles arabiensis*, exhibit a preference for breeding in open and sunlit puddles, often abundant in flooded rice fields [20]. In fact, the present study assumes that proximity to any type of irrigated agricultural crop would have the same effect on mosquito breeding. This work has not taken into account the differences between agricultural and natural wetlands. Each wetland has its own ecological characteristics, and *Anopheles* mosquito species have varying preferences for breeding habitats, which can be influenced by factors such as water quality, vegetation, sunshine and temperature. In addition, agricultural practices can introduce pesticides or alter the hydrology of the area, which can affect mosquito populations and the dynamics of malaria transmission. In addition, more intensive monitoring of the human population studied would also have enabled us to characterize the dynamics of malaria transmission over time and space, in order to better identify hotspots.

## Conclusions

In conclusion, this study investigated the transmission of malaria in an agricultural context, focusing on the influence of wetlands and agricultural practices on malaria heterogeneity in rural and semi-urban areas. The findings showed different malaria transmission patterns across rural and semi-urban sites, with higher prevalence in rural settings. Exposure to *Anopheles* mosquito bites also varied significantly within the study area. The presence of wetlands and agricultural activities, particularly irrigated crops, played a role in sustaining malaria transmission throughout the year. Proximity to wetlands increased the risk of malaria transmission in urban areas. Mapping efforts identified both micro-geographical and larger hotspot areas with persistent transmission. This study underscores the importance of considering local environmental factors for effective malaria risk assessment and management. Targeted interventions and hotspot targeting can contribute to reducing malaria incidence and facilitate malaria elimination efforts.

## Data Availability

The data that support the findings of this study are available from NMCP of Cote d’Ivoire, but restrictions apply to the availability of these data, which were used under license for the current study, and so are not publicly available. Data are however available from the authors upon reasonable request and with permission of NMCP.

## Abbreviations

ACT: Artemisinin-based combination therapy
NMCP: National malaria control program
DBS: Dried blood spots
ELISA: Enzyme-linked immunosorbent assay
HBR: Human biting rate
HLC: Human landing catches
LLIN: Long-lasting insecticidal nets
MDA: Mass drug administration
RDT: Rapid diagnostic tests
SMC: Seasonal malaria chemoprevention
